# Supplementary material for: The effect of intrapartum prolonged oxygen exposure on fetal metabolic status: secondary analysis from a randomized controlled trial
Source: Front Endocrinol (Lausanne). 2023 Jun 27;14:1204956. doi: 10.3389/fendo.2023.1204956 (PMC10335765; doi:10.3389/fendo.2023.1204956)
Supplement: Supplementary file 3 [file DataSheet_3.zip › Result-X101SC21092977-Z01-J001-B1-42/1.MetQuant-QC/Samples_QC_pos-PCA.pdf]

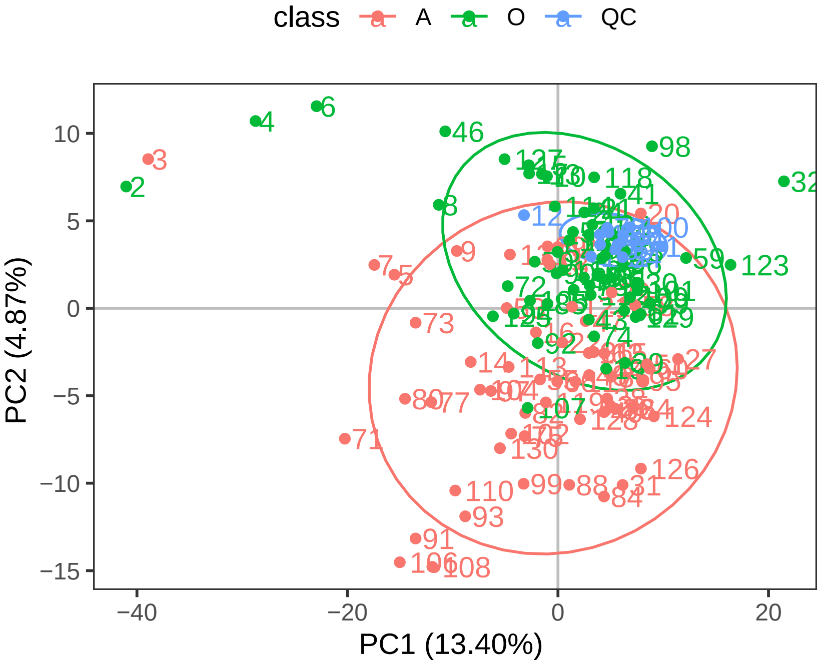

class
A
O
QC
PC1 (13.40%)
PC2 (4.87%)
−40
−20
0
20
10
5
0
−5
−10
−15
